# Suspected Pediatric Pheochromocytoma in a Normally Hypotensive Patient: Diagnostic and Management Complexity

**DOI:** 10.7759/cureus.82856

**Published:** 2025-04-23

**Authors:** Mayank Kotadia, Kayla Samimi, Aren S Saini, Jacob Gaetanos, Jane Benson

**Affiliations:** 1 Department of Anesthesiology, University of Florida College of Medicine, Gainesville, USA; 2 Department of Medicine, University of Miami Miller School of Medicine, Miami, USA; 3 Department of Radiation Oncology, University of Miami Miller School of Medicine, Miami, USA; 4 Department of Family Medicine, HCA Florida North Florida Hospital, Gainesville, USA; 5 Department of Pediatrics, Nemours Children’s Health and Wolfson Children’s Hospital, Jacksonville, USA

**Keywords:** catecholamine, chiari i malformation, neuroendocrine tumor, orthostatic hypotension, pediatric hypertension, pheochromocytoma, plasma metanephrines, secondary hypertension

## Abstract

We report the case of a 14-year-old female with a history of orthostatic hypotension and Chiari I malformation who presented with persistent hypertension, syncope, flank pain, vomiting, and back bruising and was ultimately found to have biochemical evidence of pheochromocytoma. Evaluation revealed hypercalcemia, tachycardia, and elevated plasma and urine catecholamine metabolites. Despite the absence of classic paroxysmal symptoms, such as episodic headache, palpitations, and sweating, hypertension combined with orthostatic hypotension and elevated catecholamines raised strong suspicion for pheochromocytoma. Management involved a multidisciplinary approach, with scheduled amlodipine and labetalol effectively normalizing blood pressure and heart rate. This case underscores the importance of considering pheochromocytoma in pediatric secondary hypertension, even in the absence of hallmark symptoms, and highlights possible management of overlapping orthostatic hypotension and persistent hypertension. It also demonstrates the utility of interdisciplinary care and cautious antihypertensive management in addressing the complex presentation of pediatric pheochromocytoma.

## Introduction

Pheochromocytomas are neural crest cell tumors associated with excess catecholamine production, most commonly located in the adrenal medulla. Most are benign, although malignant pheochromocytomas exist as well. Presentation can be variable, including persistent or paroxysmal hypertension, headaches, palpitations, and/or diaphoresis [1]. In the United States, pheochromocytoma is rare, with an annual incidence of only three cases per one million people, 20% of which occur in the pediatric and adolescent population [2]. This rarity makes diagnosing and managing pheochromocytoma, particularly in younger patients, especially challenging. The prevalence of pediatric hypertension has increased to 4.5%, largely due to obesity-related cases. Secondary hypertension, more common in younger children, is primarily caused by renal disease (78-80%), endocrine issues (11%), and, less frequently, by cardiac (2%) or pulmonary conditions. Pheochromocytomas and paragangliomas account for only 0.5-2% of secondary hypertension cases, so common causes should be prioritized during evaluation [3]. Symptoms, laboratory results, and family history can help identify rare catecholamine-secreting tumors.

## Case presentation

A 14-year-old female with a notable history of anxiety, Chiari I malformation, orthostatic hypotension, and unremarkable family and surgical history presented to the emergency department (ED) with pain in the lower back and bilateral lower extremities. She was found to be hypertensive with a blood pressure of 145/98 mmHg and was subsequently admitted for further evaluation and management.

The patient reported a one-day history of bilateral flank and calf pain, rated as 6/10, accompanied by two episodes of non-bloody, non-bilious vomiting. Her pain improved to 2/10 following administration of Toradol in the ED. She denied any history of trauma, joint pain, weakness, or bowel or bladder incontinence. On physical examination, a bruise was noted on her back, though it was non-tender. Additional findings included bilateral calf tenderness and tachycardia. No other significant abnormalities were identified.

Initial evaluation

Laboratory investigations revealed hypercalcemia (11.3 mg/dL), hyperproteinemia (10.1 g/dL), and proteinuria (70 mg/dL) on urinalysis (Table 1). The erythrocyte sedimentation rate was mildly elevated at 24 mm/hour, while creatine kinase and thyroid function tests were unremarkable (Table 1). Imaging with lumbar and thoracic spine X-rays showed mild spinal curvature but no acute findings.

**Table 1:** Results of additional laboratory evaluations for alternative causes of persistent hypertension.

Parameter	Result	Reference range
Iron	55 µg/dL	50–170 µg/dL
Transferrin	223 mg/dL	200–360 mg/dL
Total iron-binding capacity	332 µg/dL	250–450 µg/dL
Iron saturation	17%	20%–50%
Unsaturated iron-binding capacity	277 µg/dL	150–375 µg/dL
Vitamin D, 25-hydroxy	16 ng/mL	20–50 ng/mL
Parathyroid hormone	27 pg/mL	10–65 pg/mL
Erythrocyte sedimentation rate	24 mm/hour	0–20 mm/hour
C-reactive protein	0.1 mg/dL	<1 mg/dL
Activated partial thromboplastin	33.3 seconds	25–35 seconds
Prothrombin time-international normalized ratio	1.24	0.8–1.2
Creatine kinase	38 U/L	22–198 U/L
Serum creatinine	0.7 mg/dL	0.4–1.4 mg/dL
Total protein	10.1 g/dL	5.7–8.2 g/dL
Albumin	5.6 g/dL	3.4–5.1 g/dL
Amylase	46 U/L	23–85 U/L
Lipase	<3 U/L	22–51 IU/L
Thyroid-stimulating hormone	0.63 mIU/L	0.4–5.5 mIU/L
Serum calcium	11.3 mg/dL	8.5–10.5 mg/dL
Urine calcium, random	15.2 mg/dL	No reference range
Urine creatinine, random	54 mg/dL	No reference range
Urine sodium, random	54 mEq/L	No reference range
Urine protein	70 mg/dL	Negative
Urine protein/creatinine ratio	117 mg/g	No reference range
Aldosterone	15 ng/dL	≤35 ng/dL
Plasma renin activity	6.26 ng/mL/hour	0.25–5.82 ng/mL/hour
Aldosterone/renin ratio	2.4	0.9–28.9

**Figure 1:**
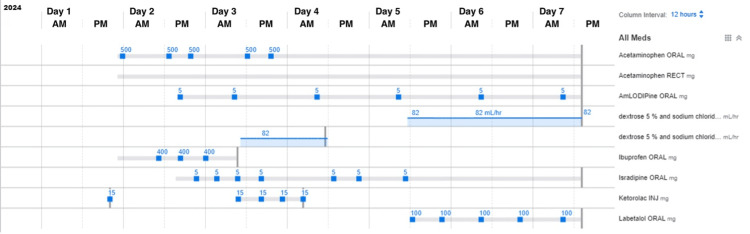
Medications with dosages administered during the seven-day hospital stay. The patient received several medications during her hospital stay. Blood pressure remained elevated despite the administration of amlodipine and isradipine but demonstrated a significant decrease coinciding with the administration of Labetalol.

The patient had a prior evaluation for orthostatic hypotension and syncope at an outside cardiology clinic in October 2024, during which she was started on midodrine 5 mg three times daily. Labs at that time included normal plasma metanephrines, catecholamines, aldosterone, and thyroid-stimulating hormone (Table 1). Given her history of orthostatic hypotension and treatment with midodrine and a high-salt diet, both interventions were held during admission due to persistent hypertension, with a maximum recorded blood pressure of 147/110 mmHg (Table 2). Initially, antihypertensive therapy was deferred out of caution for potential hypotension. However, due to sustained hypertension, the pediatric nephrology team initiated amlodipine 5 mg daily (Figure 1). Blood pressures subsequently ranged between 128-146 and 87-103 mmHg (Table 2).

**Table 2:** Hemodynamic measurements over the seven-day hospital admission. Elevated blood pressure readings indicate persistent hypertension throughout each day up until the last three days (days five to seven) when Labetalol commenced. Elevated heart rate values demonstrate a sustained increase starting on day three and a decrease at the end. Temperature remained stable around 37°C, with no evidence of fever. Note: AM readings were recorded averages for 00:00, and PM readings were recorded averages for 12:00.

	Temperature (ºC)	Heart rate (beats/minute)	Blood pressure (mmHg)	Mean arterial pressure (mmHg)
Day 1 (PM)	36.6	70	145/98	110
Day 2 (AM)	36.4	65	146/96	113
Day 2 (PM)	37	112	139/101	114
Day 3 (AM)	37.5	122	128/94	106
Day 3 (PM)	37.3	119	130/87	100
Day 4 (AM)	37.3	118	140/103	114
Day 4 (PM)	36.9	122	117/94	104
Day 5 (AM)	37.1	135	135/99	111
Day 5 (PM)	36.6	91	115/80	98
Day 6 (AM)	36.8	79	108/71	81
Day 6 (PM)	36.9	87	121/81	93
Day 7 (AM)	36.9	126	128/103	111

Further evaluation and management

**Figure 2:**
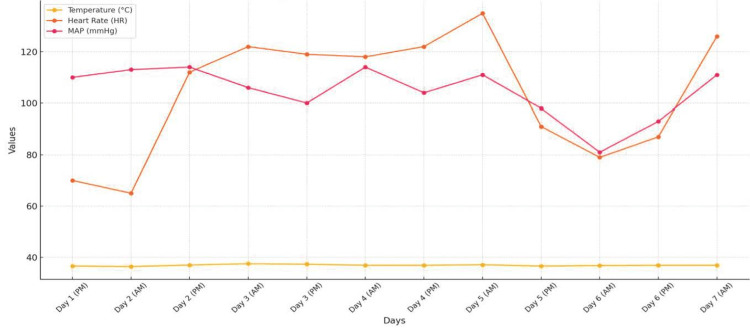
Hemodynamic trends over the course of admission.

Bilateral renal ultrasound with Doppler was unremarkable. Given clinical suspicion for pheochromocytoma, urine and plasma metanephrines, urine catecholamines, and aldosterone/renin levels were sent to an outside laboratory, with results anticipated in one week. Meanwhile, the patient’s hypertension and tachycardia persisted, with heart rates reaching 140 beats per minute. She appeared sluggish with pallor and decreased oral intake. Isradipine 5 mg every six hours as needed was initiated but produced only minimal blood pressure improvement (Figures 1, 2, Table 2).

Consultation with Pediatric Cardiology led to a transthoracic echocardiogram to rule out structural heart disease or end-organ damage from persistent hypertension. The echocardiogram was unremarkable. Additional testing revealed vitamin D deficiency (16 ng/mL) (Table 1), for which she was started on vitamin D supplementation at 2,000 IU daily. Parathyroid hormone (PTH) levels were normal (Table 1).

Given her symptoms, physical therapy was consulted to address potential deconditioning. However, the patient was unable to tolerate exercises due to tachycardia reaching 190 beats per minute. Adequate oral hydration was encouraged, and maintenance intravenous fluids (D5 ½ NS) were planned if oral intake decreased. Labetalol 100 mg twice daily was started to address both hypertension and tachycardia (Figure 1). Following this intervention, her blood pressure normalized, and her heart rate returned to within normal limits (Figure 2, Table 2). Her temperature was consistently within the normal limits, indicating a non-infectious etiology (Figure 2, Table 2).

Outcome and follow-up

The patient showed clinical improvement and was discharged in stable condition with instructions to follow up with her primary care physician within three days and the nephrology clinic within two weeks. Post-discharge laboratory results are presented in Table 3.

**Table 3:** Catecholamine profile: urine and plasma. Elevated levels of urine catecholamines, urine metanephrines, and plasma metanephrines indicate pediatric pheochromocytoma.

Parameter	Result	Reference range
Urine norepinephrine	117 µg/g creatinine	15–58 µg/g creatinine
Urine dopamine	520 µg/g creatinine	156–551 µg/g creatinine
Urine metanephrine	200 µg/g creatinine	24–302 µg/g creatinine
Urine normetanephrine	636 µg/g creatinine	14–302 µg/g creatinine
Urine total metanephrines	836 µg/g creatinine	39–578 µg/g creatinine
Plasma free metanephrine	64 pg/mL	≤57 pg/mL
Plasma free normetanephrine	597 pg/mL	≤148 pg/mL
Total free metanephrines (metanephrine + normetanephrine)	661 pg/mL	≤205 pg/mL

There were elevated fractionated random urine catecholamines, specifically, with norepinephrine at 117 µg/g creatinine (reference range: 15-58 µg/g creatinine). There were also elevated random urine metanephrines, specifically, with normetanephrine at 636 µg/g creatinine (reference range: 14-302 µg/g creatinine) and total metanephrines at 836 µg/g creatinine (reference range: 39-578 µg/g creatinine). There were elevated fractionated free plasma metanephrines, specifically, with free metanephrine at 64 pg/mL (reference range: ≤57 pg/mL) and free normetanephrine at 597 pg/mL (reference range: ≤148 pg/mL).

These findings were communicated to the family, and further imaging was recommended to evaluate for a potential adrenal mass. The patient was lost to follow-up, and additional diagnostic confirmation, such as imaging, could not be obtained.

## Discussion

Pheochromocytomas, though rare, are critical to diagnose promptly due to their potential to cause severe morbidity and mortality if left untreated. These catecholamine-secreting tumors account for approximately 0.1-0.6% of all cases of secondary hypertension [4]. This case highlights the rare presentation of secondary hypertension with a strong suspicion for pheochromocytoma in a pediatric patient with pre-existing orthostatic hypotension and Chiari I malformation. The clinical overlap between these conditions presented a diagnostic challenge, emphasizing the importance of recognizing and managing complex presentations of hypertension in adolescents. While pheochromocytoma is exceedingly rare in children, this case contributes to the growing body of evidence supporting its consideration in the differential diagnosis of pediatric secondary hypertension.

Hypertension in children is typically attributed to renal, cardiac, or endocrine causes. In this patient, the significant elevations in metanephrine and normetanephrine levels pointed strongly toward a catecholamine-secreting tumor. While hypertension is a hallmark symptom of pheochromocytoma, the presentation in this case was atypical due to the coexistence of orthostatic hypotension, syncopal episodes, and non-specific symptoms such as flank and back pain, vomiting, and bruising. The absence of the classic paroxysmal symptom triad, i.e., episodic headache, palpitations, and sweating, further complicated the clinical picture.

The concomitance of hypertension and orthostatic hypotension in pheochromocytoma is unusual and poorly understood. One hypothesis is that chronic catecholamine excess may lead to decreased vascular responsiveness due to downregulation of alpha-adrenergic receptors and impaired baroreceptor reflex function [5]. Similar cases, such as a 71-year-old male with orthostasis and syncope, have demonstrated resolution of symptoms following administration of propranolol and tumor resection [6]. However, there are very limited documented cases of such phenomena within the pediatric population. While cardiac contractility dysfunction may contribute to hypotension in some patients with pheochromocytoma, this was ruled out in our patient following a normal echocardiogram. This highlights the importance of comprehensive cardiovascular evaluation in similar cases.

The management of this case underscored the role of a multidisciplinary approach. Collaboration among the nephrology, cardiology, and physical therapy teams was crucial. Initial hesitance to initiate antihypertensive therapy due to concerns about exacerbating orthostatic hypotension was addressed via a thorough discussion between the nephrology, cardiology, and general pediatric team, leading to the selection of Labetalol, a mixed alpha- and beta-blocker. This choice was guided by evidence supporting its efficacy in lowering blood pressure steadily without causing abrupt drops [7,8]. In this case, Labetalol effectively normalized both hypertension and tachycardia, demonstrating its suitability for similar pediatric presentations.

Of note, pediatric pheochromocytomas are more often associated with germline mutations compared to their adult counterparts [2]. These mutations frequently involve syndromes such as multiple endocrine neoplasia type 2 (MEN2), von Hippel-Lindau disease, or mutations in succinate dehydrogenase (*SDHx*) genes [1]. Notably, this patient’s incidental initial hypercalcemia raises suspicion for MEN2. Further genetic testing would likely aid in discerning if there is a genetic component to the patient’s onset of symptoms; however, a significant limitation, the patient was unfortunately lost to follow-up. Nevertheless, one should consider genetic testing in pediatric cases of pheochromocytoma to identify underlying syndromic associations.

Another major limitation as a result of the patient being lost to follow-up in this case was the inability to obtain definitive imaging to confirm the presence of a pheochromocytoma. This emphasizes the critical role of robust discharge planning, patient education, and follow-up to ensure continuity of care. Future cases should prioritize streamlined transitions from inpatient to outpatient settings and clear communication with families regarding the importance of post-discharge testing and follow-up appointments to minimize diagnostic delays and optimize outcomes.

This case also serves as a reminder that rare conditions such as pheochromocytoma should remain on the differential diagnosis for pediatric hypertension, especially when initial workups for more common causes are inconclusive or unremarkable. Early identification and management can prevent severe complications, such as hypertensive end-organ damage, which was fortunately ruled out in this patient but remains a significant risk in undiagnosed cases. Clinicians managing similar cases should remain cautious of confounding factors, such as coexisting conditions or incomplete symptomatology, which may delay diagnosis. Instead, one should maintain a systematic approach to similar cases, starting with detailed history-taking and a focused physical examination, followed by appropriate laboratory and imaging studies. When pheochromocytoma is suspected, timely collection and processing of catecholamine and metanephrine levels are paramount, as delayed testing can complicate diagnosis and treatment planning.

Overall, this case underscores the challenges of managing coexisting conditions, such as orthostatic hypotension, alongside secondary hypertension, as well as possible considerations for management in such cases. The need to balance effective antihypertensive therapy while avoiding adverse effects highlights the complexity of treatment decisions. Moreover, the integration of multidisciplinary care was instrumental in achieving clinical improvement, emphasizing its value in managing such intricate presentations. By sharing this case, we aim to raise awareness of the diagnostic and management challenges of pheochromocytoma in the pediatric population.

## Conclusions

This case highlights the diagnostic and therapeutic challenges associated with pediatric pheochromocytoma, particularly when compounded by coexisting conditions such as orthostatic hypotension. It underscores the necessity of maintaining a broad differential diagnosis in cases of secondary hypertension, even in the absence of classic symptoms. Elevated catecholamine levels strongly suggested pheochromocytoma in this patient, but the lack of definitive imaging due to loss to follow-up highlights the critical importance of robust discharge planning and continuity of care. The successful management of this case, through a multidisciplinary approach and the judicious use of scheduled amlodipine and Labetalol to normalize blood pressure and heart rate, demonstrates the value of collaboration across specialties. Additionally, it emphasizes the need for systematic evaluation and consideration of genetic testing in pediatric cases. By sharing this case, we aim to improve awareness and promote early recognition and intervention for rare but life-threatening conditions such as pheochromocytoma in children.
